# Psychological Distress after the COVID-19 Pandemic among Anesthesiologists in Poland—An Observational Study

**DOI:** 10.3390/ijerph19159328

**Published:** 2022-07-30

**Authors:** Katarzyna Podhorodecka, Paweł Radkowski, Paulina Boniecka, Joanna Wojtkiewicz

**Affiliations:** 1 Students’ Scientific Club of Pathophysiologists, Department of Human Physiology and Pathophysiology, School of Medicine, University of Warmia and Mazury, 10-082 Olsztyn, Poland; paubon3434@gmail.com; 2 Department of Physiology, School of Medicine, Collegium Medicum, University of Warmia and Mazury, 10-082 Olsztyn, Poland; joanna.wojtkiewicz@uwm.edu.pl; 3 Department of Anaesthesiology and Intensive Care, School of Medicine, Collegium Medicum, University of Warmia and Mazury, 10-561 Olsztyn, Poland; pradkowski@wp.pl; 4 Department of Anaesthesiology and Intensive Care, Regional Specialist Teaching Hospital, 10-561 Olsztyn, Poland

**Keywords:** COVID-19, pandemic, anesthesiologists, burnout, well-being, stress

## Abstract

Introduction: The response to the COVID-19 pandemic by anesthesiologists has been simply heroic. Unfortunately, there are very few evidence-based studies in the literature that focus on anesthesiologists’ burnout during that time. The purpose of our study was to examine the psychological distress, after the COVID-19 pandemic, among anesthesiologists in Poland. Methods: We conducted an anonymous internet survey among a group of anesthesiologists in Poland. It contained a questionnaire, entitled “Oldenburg Burnout Inventory (OLBI)”, with demographic questions about sex, age, and family, as well as questions related to working conditions during the COVID-19 pandemic. We received data from 158 people, including 109 women and 49 men. Results: Results from the analysis showed that 73% (115/158) of the participants suffered from burnout. Moreover, 95.6% of the participants thought that the COVID-19 pandemic had had an influence on their level of burnout, and 97.3% found that it had had a negative impact. Conclusions: There is no doubt that healthcare workers, despite the difficulties associated with their daily work, have not faced challenges on such a scale in a very long time. Support for their mental health should be an essential component of the modern public healthcare system.

## 1. Introduction

The first case of a patient infected with SARS-CoV-2 dates back to December 2019 and was first reported in Wuhan (China). Since then, the virus has spread around the world, causing a huge, unprecedented crisis in global healthcare. As of today (April 2022), global COVID-19 deaths exceed 6 million, and confirmed cases have reached 505 million [1]. The high infectivity rate of this disease and its associated mortality and morbidity have created fear among the general population, causing social dysfunction, mass hysteria, and a constant state of anxiety. The severity of the complications of this disease and its high mortality rates have resulted in patients needing to be hospitalized to receive specialized care, which has acutely taxed the limits of healthcare systems.

Faced with this critical situation, frontline healthcare workers directly involved in the treatment and care of patients with SARS-CoV-2 infection are at risk of developing psychological distress and other mental health symptoms. Increasing numbers of confirmed or suspected cases; an overwhelming workload; widespread media coverage; a lack of specific drugs; and feelings of being insufficiently supported, of vulnerability, or of loss of control may all have contributed to the mental burden on these workers. They experienced one of the great paradoxes of the COVID-19 pandemic: while the general population had to remain at home and avoid any social contact, healthcare professionals had to continue to perform their work in direct contact with the virus and be continually exposed to it.

Of the more than 5.5 million cases of SARS-CoV-2 infection confirmed in Poland since the outbreak began, 45,705 involved doctors. Coronavirus in Poland—as reported by the Ministry of Health—has contributed to the deaths of 304 doctors [2]. Unfortunately, there are no accurate statistics on how large a percentage of these physicians were anesthesiologists. Initially, the biggest concern was the lack of personal protective equipment (PPE) related to COVID-19, but then there was also a shortage of disposable gowns, masks, and pads necessary for the daily operation of surgical, oncology, intensive care, and transplant units.

The situation of the market was bizarre, with disposable items reaching prices that were impossible for state-owned facilities to cover. Changes in regulations, the obligation to wear masks, and a huge increase in the demand for these articles caused the price of needed supplies to become too expensive for hospitals. Disposable masks were sewn by volunteers and even by prison inmates. However, these did not meet any relevant standards.

Anesthesiologists’ response to the pandemic has been nothing short of amazing. They constitute a distinct group of frontline healthcare personnel who have faced significant problems during the pandemic. Being experts in airway management, critical care management, and the resuscitation of patients, anesthesiologists are playing a major role in the COVID-19 pandemic. As a result of their participation in multiple aerosol-producing procedures, such as pre-oxygenation, mask ventilation, laryngoscopy, tracheal intubation, the suctioning of orotracheal secretions, and extubation, they were highly susceptible to becoming infected, especially if adequate personal protective equipment (PPE) was not available [3].

Previous studies have shown that doctors struggled with anxiety, depression, insomnia, burnout, and significant psychological distress during the COVID-19 outbreak [4,5,6]. Frontline work was an independent factor in worse mental health outcomes across all dimensions of interest [7]. Burnout, a syndrome related to work and characterized by emotional exhaustion, low personal fulfillment, and depersonalization, is already widespread among anesthesiologists. Prevention is crucial, as burnout has important implications for both institutions and individuals; therefore, the routine assessment and measurement of burnout among team members by leadership is an essential intervention [8]. However, we have a poor understanding of their psychological discomfort, and there are very few evidence-based studies in the literature that focus on anesthesiologists’ burnout during that time that would allow us to intervene in a timely and effective manner [9].

The aim of our study was to examine the psychological distress after the COVID-19 pandemic among anesthesiologists in Poland.

## 2. Material and Methods

In 2022, we conducted an anonymous internet survey among a group of anesthesiologists in Poland. This questionnaire was entitled “Oldenburg Burnout Inventory (OLBI)”, and contained demographic questions about sex, age, and family, as well as those related to working conditions during the COVID-19 pandemic. For this study, we chose the OLBI test because it has good psychometric parameters. This tool is characterized by a good internal consistency and accuracy. It also has good enough reliability indices [10]. The link to the survey was shared on social media pages that targeted anesthesiologists across the country.

The number of physicians with a specialization in anesthesiology and intensive care in Poland is approximately 4000. The total number of anesthesiologists-intensivists, including those with incomplete specialization, is almost 7000 [11]. We received data from 158 people, including 109 women and 49 men. This difference reflected, to some extent, the gender ratio of the medical profession in Poland. Currently in Poland, women constitute more than 60% of physicians actively practicing this profession [11]. Based on the information derived from the previous article about the OLBI questionnaire [12], participants were divided into four groups: burnout, exhausted, disengaged, and non-burnout. Each group was categorized depending on the mean score from the OLBI test. The OLBI test contains 16 items. Items 2(R), 4(R), 5, 8(R), 10, 12(R), 14, and 16 measure exhaustion, while 1, 3(R) 6(R), 7, 9(R), 11(R), 13, and 15 measure disengagement. Each item is scored: strongly agree—1 point; agree—2 points; disagree—3 points; and strongly disagree—4 points. The scores were reversed in the items with the letter “R”. The higher the scores are, the greater the exhaustion and disengagement are [12].

*Categories*:

1. Burnout group: high exhaustion mean scores of ≥2.25 and high disengagement mean scores of ≥2.1.
2. Exhausted group: high exhaustion mean scores of ≥2.25 and low disengagement mean scores of <2.1.
3. Disengaged group: high disengagement mean scores of ≥2.1 and low exhaustion mean scores of <2.25.
4. Non-burnout group: low disengagement mean scores of <2.1 and low exhaustion mean scores of <2.25.

For the statistics measure, we used the following tests: a Wilcoxon test, chi-square test, and Pearson correlation test. The level of statistical significance was determined by a *p*-value of <0.05.

## 3. Results

Results from the analysis showed that 73% (115/158) of participants suffered from burnout (Table 1). The average age of all the participants was 38 years. The mean age of a burned person was 38, and the mean age of people without burnout was 39. 

The most common issues that had an impact on anesthesiologists’ low motivation to work were: non-compliance with sanitary and epidemiological recommendations by others (117/158, 74%), poor organization of care for patients suffering from COVID-19 (106/158, 67%), a high number of working hours (95/158, 60%), high levels of stress (91/158, 58%), and a low salary (46/158, 29%). The most common trigger stressors were: not seeing the effects of intensive care treatment (130/158, 82%), many hours wearing personal protective equipment (96/158, 61%), and the unpredictable course of this disease (82/158, 52%).

The average time of working was 245 h per month. In total, 54% (86/158) of participants worked more during the COVID-19 pandemic than they had before. A total of 98% (155/158) of the participants were vaccinated against SARS-CoV-19. In total, 54% (85/158) of participants contracted COVID-19. In total, 91% (144/158) of the participants were satisfied with their choice of specialization. A total of 96% (152/158) of the participants thought that the COVID-19 pandemic had had an influence on the level of burnout among doctors, and 97% (153/158) of them believed that it had had a negative impact. In total, 61% (96/158) of the participants believed that the salary supplements for working with COVID-19 patients did not increase their motivation to work. Participants rated the work organization during the pandemic at the hospital with an average score of 4.5 out of 10.

The results (Table 2) indicated that burned-out participants statistically played less sport than the group without burnout (24% vs. 40%; *p* = 0.045).

Statistically, the non-burnout group took part in physical activity more often than the burnout group: 40% vs. 24%. Occupational burnout (burnout vs non-burnout group) was not statistically influenced by: having children 62% vs. 67%, being a specialist 62% vs. 61%, being a resident 38% vs. 40%, being a woman 67% vs. 74%, being a man 33% vs. 26%, working more than 15 years 24% vs. 35%, or working less than 15 years 77% vs. 65%. At the limit of the statistical significance, more people in the burnout group (compared to the non-burnout group) indicated that they changed their working place during the pandemic (40% vs. 23% *p* = 0.05019). Our analysis also showed that, at the limit of the statistical significance, more people in the burnout group work in a city with more than 100,000 residents (85% vs. 72% *p* = 0.057).

We also asked our respondents to indicate on a scale of 1 to 10 (where 10 meant the highest level of stress), how they would rate their level of stress at work before and during the pandemic. Using the Wilcoxon test (Table 3), we compared two dependent groups: pre-pandemic stress levels at work and stress levels at work during the COVID-19 pandemic. Our study also indicated that during the pandemic, the median level of stress was statistically higher (9/10) than the level of stress at work before (6/10). 

There was a statistically significant *p* = 0.003 negative correlation in the Pearson correlation test of r = −0.234 between the number of working hours and the results in the OLBI test. The results showed that if the participant was burned out, they were working less. 

## 4. Discussion

The working conditions of anesthesiologists in Poland have long raised many doubts. The ongoing pandemic has only worsened this situation. According to the audit carried out by the Supreme Chamber of Control in Poland, in some cases, anesthesiologists work 78–125 h without a break, which could adversely affect the quality of their tasks and pose a threat to patients and the physicians themselves [13]. Studies in other countries have shown that up to one in two anesthesiologists are at risk of burnout syndrome [14]. In our study, as many as 73% of anesthesiologists were classified as burned out based on the OLBI test. This is truly astonishing considering the fact that 91.1% of the participants were satisfied with their choice of specialization. Previous studies describing the psychological impact of the COVID-19 pandemic on anesthesiologists showed that being under 35 years of age, being a female, one’s marital status, being a resident doctor, the fear of infecting oneself or one’s family, the fear of salary deductions, an increase in working hours, loneliness caused by isolation, food and accommodation issues, and postings in COVID-19 duty were risk factors for anxiety [3]. However, in our survey of Polish anesthesiologists, we found no correlation between burnout and gender, age, having a child, or being a resident or a specialist. Nevertheless, we discovered that burned-out participants statistically played less sport than the group without burnout. This is consistent with another study that found regular sports participation to be a factor in reducing burnout [15].

In addition to the psychological aspects of the pandemic on society, healthcare workers were under stress due to their remarkable risk of infection, lack of personal protective equipment, fear of spreading the infection to family members, working under extreme pressure, social isolation, economic consequences, and direct involvement in treating infected patients. Furthermore, they were exposed to a high level of suffering from the patients they cared for in an overwhelmed healthcare system, which was aggravated by uncertainty about the disease, the severity of the symptoms, and the fear of death of the patients as an actual possibility. Participation in aerosol-dispensing procedures and frequent contact with patients increased the risk of infection, which can also be a concern for staff living with the elderly and young children. Our study showed a correlation between the number of working hours and burnout. The results showed that if the participant was burned out, they were likely to be working less. This can increase the burden on other employees, which in turn can be a contributing factor to burnout. This is a perfect example of a vicious cycle.

The participants also struggled with sensitive and unusual responsibilities and were part of the decision-making process for emotionally draining decisions, such as involvement with grieving families or with families that were no longer allowed to visit their loved ones. Previous findings have indicated that the COVID-19 pandemic is associated with a high incidence of generalized anxiety disorder and an increased sense of burnout among critical care anesthesiologists, especially in females and younger physicians [16,17]. In addition, long hours spent in PPE, exhausting prolonged working hours, and stress related to the uncertainty of the outbreak could all lead to anxiety, depression, insomnia, and post-traumatic stress disorder [3]. During the COVID-19 pandemic, anesthesiologists encountered an unprecedented phenomenon. Through the difficulties of performing procedures with masks, visors, and steaming goggles, doctors had to learn how to perform procedures all over again. Their experiences did not matter here. Another stressful component was talking to patients about their unfavorable prognoses, especially since these conversations were conducted in personal protective equipment, which made communication difficult. From our experience, and also from the responses of the interviewees, the procedures that aroused particularly high levels of stress were intubation, after which it took a long time for the saturation level to rise, as well as procedures such as the prone positioning of the patient. We also compared stress levels from before the pandemic to those during the COVID-19 outbreak. The results indicated that during the pandemic, the median level of stress was statistically higher (9/10) than the level of stress at work before (6/10).

In our study, the participants claimed that non-compliance with sanitary and epidemiological recommendations by others, the poor organization of care for patients suffering from COVID-19, a high number of working hours, high levels of stress, and low salaries were the most important factors that affected their motivation to work. Another interesting finding was that, although Poland is one of the few countries with salary supplements for working with COVID-19 patients, as many as 29% of respondents indicated low salary as a factor that decreased their motivation to work. Moreover, 60.7% of the participants believed that these salary supplements did not increase their motivation to work. This may indicate that the working conditions were so dramatic that no financial compensation could have made up for it. Additionally, they believed that the most common trigger stressors were: not seeing the effects of intensive care treatment, many hours in personal protective equipment, and the unpredictable course of this disease. Addressing those factors could improve their psychological well-being and their working outcomes.

Previous studies have indicated that factors with the potential to reduce anxiety are: clear communication, limited shift hours, the availability of rest areas, broad access to and detailed rules on the use and management of protective equipment, and specialized training on handling COVID-19 patients [6].

We should tailor the treatment of burnout to its level. In the beginning, it may be sufficient to change life habits or introduce a balance between work and life. Previous studies have shown that this could be achieved by spending quality time with family, friends, and significant others, as well as with relaxation and sport [15]. We should also consider giving physicians the ability to influence their work environment. If more severe cases of burnout occur, we may also consider psychotherapy or anti-depressant treatment combined with psychotherapy [15].

We must keep in mind that our study had some limitations. The first limitation was the small number of participants. Additionally, we could not control for every possible lifestyle factor, and the observational nature of this design left the possibility of residual confounding. Moreover, we had no data that would reliably show what the mental health of Polish physicians was like. Those that we had were mostly based on voluntary questionnaires. Therefore, we only had answers from those who were ready to give them, and these contained only the content that they decided to disclose.

## 5. Conclusions

Anesthesiology is undoubtedly a specialty in which we deal with the sickest patients, or with healthy patients to whom we administer drugs that can cause adverse effects in a short period of time. This situation has certainly been exacerbated by the COVID-19 pandemic. There is no doubt that healthcare workers, despite the difficulties associated with their daily work, have not faced challenges on such a scale in a very long time. Support for their mental health should be an essential component of the modern public healthcare system. Our report showed that the situation in Polish anesthesiology is dramatic. In our study, as many as 73% of anesthesiologists were classified as burned out based on the OLBI test. It is well known that in most hospitals throughout Poland there is a shortage of physicians, especially specialists. Introducing work standards should be a key step for employees, as well as for management. Fatigue, lack of sleep, and time pressure increase the incidence of medical errors and make it challenging to provide adequate patient care [15]. Due to uncertainty about the duration of the current pandemic, one can only assume that the impact will be considerable. Therefore, addressing the issues leading to increased burnout is important in order to reduce the long-term negative consequences. 

## Figures and Tables

**Table 1:** Division participants in groups, depending on the mean scores derived from the OLBI test.

Variable	Frequency n = 158	Percent %
Non-burnout group	7	4%
Disengaged group	19	12%
Exhausted group	17	11%
Burnout group	115	73%

**Table 2:** **Summary descriptive table compares the proportions of burnout vs. non-burnout according to the given questions**. *p*-values were measured using a chi-square test. We measured whether factors, from the questions asked, statistically influenced being burned out in an anesthesiologist’s job. We considered whether playing sports, having a child, being a specialist or a resident, being a female or a male, the number of working hours, working less or more than 15 years, changing jobs during the COVID-19 pandemic, or living in a city of more or less than 100,000 people affected the risk of professional burnout.

Questions from Survey	Groups	Burnout Group n = 115	Exhausted, Disengaged, and Non-Burnout Groups. n = 43	*p*-Value Compare Factors vs. Being Burnout
Does the participant play sports?	Yes 44/158 (28%)	27/115 (24%)	17/43 (40%)	0.045 (<0.05) Playing sport does affect being burnout.
	No 114/158 (72%)	88/115 (77%)	26/43 (61%)	
Does the participant have a child?	Yes 100/158 (63%)	71/115 (62%)	29/43 (67%)	0.508 (>0.05) Having child does not affect being burnt out.
	No 58/158 (37%)	44/115 (38%)	14/43 (33%)	
Does the participant is a specialist or a resident of anesthesiology?	Specialist 97/158 (61%)	71/115 (62%)	26/43 (61%)	0.883 (>0.05) Being a specialist or resident does not affect being burnt out.
	Resident 61/158 (39%)	44/115 (38%)	17/43(40%)	
What is the gender of the participant?	Female 109/158 (69%)	77/115 (67%)	32/43 (74%)	0.361 (>0.05) Being female or male does not affect being burnout.
	Male 49/158 (31%)	38/115 (33%)	11/43 (26%)	
How many years has the participant been working?	Longer than 15 years 42/158 (27%)	27/115 (24%)	15/43 (35%)	0.155 (>0.05) Working longer than 15 years or less than 15 years does not affect being burnt out.
	Less than 15 years 116/158 (73%)	88/115 (77%)	28/43 (65%)	
Did the participant change the place of work during COVID-19?	Yes 56/158 (35.4%)	46/115 (40%)	10/43 (23%)	0.05019 (>0.05) Changing the place of work during COVID-19 does not affect being burnt out.
	No 102/158 (65%)	69/115 (60%)	33/43 (77%)	
How many inhabitants are there in the city in which the participant works?	More than 100 thousand 129/158 (82%)	98/115 (85%)	31/43 (72%)	0.057 (>0.05) Living in the city with more than 100,000 inhabitants or less than 100,000 inhabitants does not affect being burnt out.
	less than 100 thousand 29/158 (18%)	17/115 (15%)	12/43 (28%)	

**Table 3:** Comparing the median in two dependent groups by Wilcoxon test.

	Pre-Pandemic Stress Levels at Work	Stress Levels at Work During the COVID-19 Pandemic	The *p*-Value in the Wilcoxon Test
Median (on scale from 1 to 10)	6	9	*p* = 0.0000 (*p* < 0.05)

## Data Availability

The data presented in this study are available on request from the corresponding author.
